# Consensus genomic regions for grain quality traits in wheat revealed by Meta‐QTL analysis and in silico transcriptome integration

**DOI:** 10.1002/tpg2.20336

**Published:** 2023-05-05

**Authors:** Na Li, Yongping Miao, Jingfu Ma, Peipei Zhang, Tao Chen, Yuan Liu, Zhuo Che, Fahimeh Shahinnia, Delong Yang

**Affiliations:** ^1^ State Key Laboratory of Aridland Crop Science Gansu China; ^2^ College of Life Science and Technology Gansu Agricultural University Gansu China; ^3^ Plant Seed Master Station of Gansu Province Gansu China; ^4^ Institute for Crop Science and Plant Breeding, Bavarian State Research Centre for Agriculture Freising Germany

## Abstract

Grain quality traits are the key factors that determine the economic value of wheat and are largely influenced by genetics and the environment. In this study, using a meta‐analysis of quantitative trait loci (QTLs) and a comprehensive in silico transcriptome assessment, we identified key genomic regions and putative candidate genes for the grain quality traits protein content, gluten content, and test weight. A total of 508 original QTLs were collected from 41 articles on QTL mapping for the three quality traits in wheat published from 2003 to 2021. When these original QTLs were projected onto a high‐density consensus map consisting of 14,548 markers, 313 QTLs resulted in the identification of 64 MQTLs distributed across 17 of the 21 chromosomes. Most of the meta‐QTLs (MQTLs) were distributed on sub‐genomes A and B. Compared with the original QTLs, the confidence interval (CI) of the MQTLs was smaller, with an average CI of 4.47 cM, while the projected QTLs CI was 11.13 cM (2.49‐fold lower). The corresponding physical length of the MQTL ranged from 0.45 to 239.01 Mb. Thirty‐one of these 64 MQTLs were validated in at least one genome‐wide association study. In addition, five of the 64 MQTLs were selected and designated as core MQTLs. The 211 quality‐related genes from rice were used to identify wheat homologs in MQTLs. In combination with transcriptional and omics analyses, 135 putative candidate genes were identified from 64 MQTL regions. The findings should contribute to a better understanding of the molecular genetic mechanisms underlying grain quality and the improvement of these traits in wheat breeding.

## Core Ideas

- We collected 508 quantitative trait loci (QTLs) associated with quality traits in wheat and performed meta‐QTL (MQTL) analysis.
- We identified 64 MQTLs, 31 of which were validated by marker‐trait association in genome‐wide association studies, and five were selected as core MQTLs.
- Through the comprehensive platform, 1219 important candidate genes were excavated.
- Through transcriptomics and gene function analysis, 135 highly expressed genes were identified.
- This study aims to integrate QTL for quality traits in wheat and identify important genomic regions and key genes.

**Abbreviations**
BLASTN: basic local alignment search tool
BLASTP: basic local alignment search tool for proteins
CI: confidence interval
DART: diversity arrays technology
FPC: flour protein content
GPC: grain protein content
GWAS: genome‐wide association study
LOD: logarithm of the odds
MQTL: meta‐QTL
MTA: marker‐trait association
PC: protein content
QTL: quantitative trait loci
SNP: single nucleotide polymorphism
SSR: simple sequence repeat
TPM: transcripts per million
TW: test weight
WGC: wet gluten content

## 1 INTRODUCTION

Wheat (*Triticum aestivum* L.) is one of the most important food crops worldwide, providing about 20% of the calories, protein, and fiber for the global human diet (Asseng et al., 2011; Ling et al., 2013; Shiferaw et al., 2013; Yadav et al., 2019), and its economic value has also been determined by the intrinsic grain quality factors affecting the final products (Ammiraju et al., 2001). Therefore, improving quality traits has long been an important goal in wheat breeding (Deng et al., 2013; Jin et al., 2016; Nelson et al., 2006). Due to the importance of protein and gluten content, test weight (TW) for bread production, nutritional value, and economic impact of end product quality, wheat quality breeding has also mainly focused on improving these essential components (Liu et al., 2017; Ruan et al., 2021; Sun et al., 2009).

Grain quality is a complex quantitative trait that is largely influenced by genetic traits, ecological factors, and cultivation practices (Guo & Wang, 2006; Li et al., 2005; Quraishi et al., 2017; Souza et al., 2004; Zhang et al., 2004). Stem length, plant height, spikelet length, flowering date, and several yield traits have been reported to affect quality traits (Jiang et al., 2019). Number of spikelets, grains per spike, thousand grain weight, and grain yield are significantly and negatively correlated with some quality traits of wheat (Yang et al., 2020). Identification of important quantitative trait loci (QTLs) and further utilization of superior genes are essential for genetic improvement of grain quality in modern breeding programs. Over the past two decades, numerous studies using traditional biparental linkage mapping (Groos et al., 2003; Krishnappa et al., 2021; Ma et al., 2012) and genome‐wide association studies (GWAS) (Fiedler et al., 2017; Gao et al., 2021; Lou et al., 2021; Sallam et al., 2020) have been conducted for grain quality traits in wheat. QTLs for protein content (Blanco et al., 2002; Huang et al., 2006; Prasad et al., 2003; Yang et al., 2013) and TW (Mccartney et al., 2005; Sun et al., 2010, 2009; Yang et al., 2013) have been reported on all chromosomes, while QTLs for gluten content have been reported on all chromosomes except chromosome 3D (El‐feki et al., 2013; Li et al., 2009; Ma et al., 2012). Most of these QTLs contribute nearly 50% to explaining phenotypic variations. However, due to differences in genetic background of populations, genetic map, marker types, and environmental conditions, many of the previously identified QTLs are of low accuracy and have no direct application value in wheat molecular breeding (Kumar et al., 2020; Liu, Mullan et al., 2020; Yang et al., 2007).

**Core Ideas**

- We collected 508 quantitative trait loci (QTLs) associated with quality traits in wheat and performed meta‐QTL (MQTL) analysis.
- We identified 64 MQTLs, 31 of which were validated by marker‐trait association in genome‐wide association studies, and five were selected as core MQTLs.
- Through the comprehensive platform, 1219 important candidate genes were excavated.
- Through transcriptomics and gene function analysis, 135 highly expressed genes were identified.
- This study aims to integrate QTL for quality traits in wheat and identify important genomic regions and key genes.

Meta‐analysis is a method of integrating QTLs obtained in previous studies to perform more comprehensive and accurate experiments for improvement of complex quantitative traits (Goffinet et al., 2000; Hanocq et al., 2007; Sosnowski et al., 2012; Veyrieras et al., 2007). This method has been successfully and widely used in plant breeding (Chardon et al., 2004; Wang et al., 2013), and has achieved good results in QTL integration of various quantitative traits in several crops, for example, yield‐related traits of maize (*Zea mays* L.) (Chen et al., 2017), insect‐resistant combinations in maize (Chen et al., 2017), yield‐related traits in rice (*Oryza sativa* L.) (Bhatia et al., 2017), and quality traits of cotton (*Gossypium hirsutum* L.) (Adhikari et al., 2017). In addition, useful results have been reported from meta‐QTL (MQTL) studies on several related traits in wheat, such as yield‐related (Xu et al., 2017) traits, grain quality traits (Jernigan et al., 2017), root‐related traits (Soriano et al., 2019), and disease resistance (Liu, Salsman et al., 2020; Soriano et al., 2015; Venske et al., 2019). However, most of these studies did not examine the candidate genes behind the MQTLs, because the reference sequence of the wheat genome was not available in the past. With the advent of the high‐quality Chinese spring wheat reference genome reported by the International Wheat Genome Sequencing Consortium (International Wheat Genome Sequencing Consortium [IWGSC] et al., 2018) and a large amount of wheat transcriptome data on a user‐friendly platform (Borrill et al., 2016; Ramírez‐González et al., 2018), meta‐analysis has recently been used to identify key candidate genes, opening unexpected opportunities for breeding. Many key genes cloned by homology‐based methods have been confirmed in yield‐related MQTL regions (Yang et al., 2021), such as *TaVrn1* (Yan et al., 2003), *TaVrn2* (Yan et al., 2004), *TaVrn3* (Yan et al., 2006), *TARHT‐B1* (*Rht1*) (Peng et al., 1999), two copies of *TaPpd* (Beales et al., 2007), *Tags‐D1* (Zhang et al., 2014), *TaCKX2* (Zhang et al., 2011), and *TaTGW6* (Hanif et al., 2016). However, in wheat, only a few major quality genes have been isolated by map‐based cloning, so understanding the molecular basis of QTL/genes controlling grain quality still lacks essential knowledge.

In this study, the major genomic regions and putative candidate genes for grain quality traits in wheat were identified through meta‐analysis. The objective of this study was to integrate the genomic regions associated with previously published QTLs for quality traits to obtain MQTLs with smaller confidence intervals (CIs) and transcriptome evidence for candidate gene identification, which will help improve our understanding of the genetic basis controlling grain quality. These reliable QTLs and candidate genes can then be used in further breeding research to improve wheat quality.

## 2 MATERIALS AND METHODS

### 2.1 Literature search and QTL data collection

The literature on QTL mapping of grain quality traits including protein content (PC), grain protein content (GPC), flour protein content (FPC), wet gluten content (WGC), and TW was collected from the X‐MOL academic platform (https://www.x‐mol.com/), the literature repository (http://459.org/), and the Chinese National Knowledge Internet (https://www.cnki.net/) using appropriate keywords (QTL, quality trait, and wheat). For each QTL, the following data were collected: (1) associated trait; (2) population type of QTL mapping; (3) population size; (4) LOD values; (5) phenotypic variance explained (PVE) values; and (6) flanking or closely related markers. If the LOD and PVE values were missing, they were assumed to be 3% and 10%, respectively, according to the method proposed by Li et al. (2010) and Khahani et al. (2020). When the CI of the QTL was missing, the CI of 95% was calculated according to population type, size, and PVE. The standard formula was given as follows: (1) F_2_ population, CI = 530/(N × PVE); (2) recombinant inbred line (RIL) population, CI = 163/(N × PVE); (3) double haploid (DH) population = 287/(N × PVE) (Darvasi et al., 1997; Guo et al., 2006; Visscher et al., 2004; Yang et al., 2021). Number of lines was the size of the mapping population used for QTL analysis, and PVE was the phenotypic variance explained by QTL.

### 2.2 Initial QTL projection and MQTL analysis

After initial QTL data collection, all individual QTLs were projected onto a reference map by using BioMercator v4.2.3 software (Sosnowski et al., 2012). The high‐density reference map developed by Bilgrami et al. (2020) was used, which is an integration of genetic reference maps from two previously published genetic maps (Maccaferri et al., 2015; Soriano et al., 2019). This map contains 14,548 markers, including simple sequence repeat (SSR), diversity arrays technology (DART), and single nucleotide polymorphism (SNP), and other types of markers, with a total length of 4813.72 cM and a marker density of 3.02/cM. The QTLs that could not be found on the consensus map were eliminated. On each chromosome, the MQTL were calculated using the following two methods. In the first method, the meta‐analysis proposed by Goffinet et al. (2000) was applied when the number of original QTLs on a chromosome was less than 10. Based on this approach, the best MQTL model with the lowest Akaike information criterion (AIC) values for QTL integration and identification of consensus MQTL positions in BioMercator was selected. In the second method, if the number of QTLs in a chromosome was at least 10, a two‐step approach proposed by Veyrieras et al. (2007) was used. In the first step, the collected QTLs on individual chromosomes were clustered using standard parameters. The number of potential MQTLs per chromosome were then estimated based on the following five selection criteria, including AIC, Akaike information criterion correction (AICc), Akaike information criterion 3 candidate models (AIC3), Bayesian information criterion, and approximate weight of evidence. A QTL model that had the lowest values of the selection criteria was considered the best optimal model for the next step of meta‐analysis. In the second step, the 95% CI and the positions of each MQTL were determined according to the optimal model selected in the previous step. The QTLs were integrated so that the peak position of the original QTLs was in the MQTL CI (Daryani et al., 2022), whereas the QTLs without the minimum AIC values were to be discarded.

### 2.3 Delineation of MQTL physical positions and verification by GWAS

To determine the physical positions of all MQTLs, the flanking markers within the target MQTLs were first found through a manual search based on the 95% CIs and the location of each MQTL. Then, flanking target MQTL markers were searched using the “Marker Information” function of the Triticeae Wheat Multi‐Omics Center (http://wheatomics.sdau.edu.cn) to find the physical positions. If the physical positions of the flanking marker were not found, the GrainGenes database (https://wheat.pw.usda.gov/GG3) or the DART database (https://www.diversityarrays.com) were used to search for flanking marker sequences. The most likely physical positions were identified using the BLASTN program based on the Chinese spring chromosome RefSeq V1.0 from the Chinese Triticeae Multi‐omics Center.

Quality trait data from 13 GWAS published from 2017 to 2021 were collected and used to verify the accuracy of these MQTL regions. Phenotypic data from these studies were collected with population sizes ranging from 163 to 2038, including spring wheat populations, winter wheat populations, and mixed populations of spring and winter wheat. Similarly, the physical position of marker‐trait associations (MTAs) in these studies was determined using the source papers or BLASTN of the flanking marker sequences (Gudi et al., 2022; Yang et al., 2021) and compared with the physical coordinates of MQTLs (chromosome‐wise). A single MTA falling within the genomic region of one or more MQTLs was considered co‐localized (Saini et al., 2022).

### 2.4 Search for putative genes in the MQTL regions

Putative genes in the MQTL region were identified by the following three methods: (1) To identify the major putative candidate genes in the MQTL region using the strategy of orthologous wheat‐rice comparison, the basic information on all functionally verified putative genes related to rice grain quality was downloaded from China Rice Data Center (https://www.ricedata.cn/). Based on IWGSC RefSeqv1.1 (http://wheat.cau.edu.cn/TGT), the homologous genes in wheat were found using Triticeae‐Gene Tribe (http://wheat.cau.edu.cn/TGT). The putative genes located in the MQTL regions were considered as important candidate genes affecting grain quality (Yang et al., 2021). (2) To obtain the core MQTL, the selection criteria (Venske et al., 2019) were as follows: (a) at least two overlapping QTL projections must be present in the resulting MQTL; (b) physical distance of less than 20 Mb; and (c) genetic distance of less than 1.0cM for each MQTL interval. To this end, high‐probability genes within each highly refined MQTL were listed and then designated as putative candidate genes using the Triticeae Multi‐omics Center (http://wheatomics.sdau.edu.cn) based on genomic features annotated by IWGSC_v1.1_HC_gene. (3) For the remaining MQTLs with longer CI, only a genomic region of 2 Mb (1 Mb region on both sides of the MQTL peak) was analyzed for the occurrence of putative candidate genes (Saini et al., 2022). The physical position of the peak of the MQTLs was calculated according to the method proposed by Saini et al. (2022).

The “Genes” tool of the WheatGmap database (https://www.wheatgmap.org/tools/gene/information) was used to obtain detailed information (locus ID information and functional description) about the gene models in the MQTL regions detected by the last two methods. In addition, known wheat genes associated with the quality trait were searched for, and their physical positions were determined by the Wheat Multiomics Center (http://wheatomics.sdau.edu.cn) using their available IDs from previous corresponding studies. The physical positions of these genes were then compared to the genomic MQTL regions to determine their co‐localizations (Saini et al., 2021).

### 2.5 Expression analysis of putative genes in the MQTL regions

Functional analysis of all genes from the MQTL region was performed using gene ontology (GO) enrichment on the GENEDENOVO cloud platform (https://www.omicshare.com/tools/) Then, Kyoto Encyclopedia of Genes and Genomes (KEGG) pathway enrichment analysis (https://www.omicshare.com/tools/) was used to filter out some metabolic pathways of all genes, such as starch and sucrose metabolism, protein processing, and so on. Finally, all genes available from the MQTL regions were subjected to further expression analysis using an Expression Visualization and Integration Platform (expVIP, http://www.wheat‐expression.com) (Borrill et al., 2016), which includes expression data from 18 tissues throughout the wheat growth period (Ramírez‐González et al., 2018). The expression levels of the putative candidate genes were assessed using at least two transcripts per million (TPM) and displayed using the TBtools (https://github.com/CJ‐Chen/TBtools/releases) of TPM based on the normalized scale method. The transcriptomic data (http://www.wheat‐expression.com) in this study included five tissues at different growth stages, such as grain at 2 dpa (day post anthesis), 14 dpa, and 30 dpa stages; spike at two nodes detectable, flag leaf stage and anthesis; leaf (excluding flag leaf) at tillering stage, 2 dpa and sowing stage; stem at 1 cm spike, two nodes detectable, and anthesis stage; roots at seedling stage, three leaf stage, and flag leaf stage.

## 3 RESULTS

### 3.1 Grain quality trait characteristics obtained from QTL studies

**FIGURE 1:**
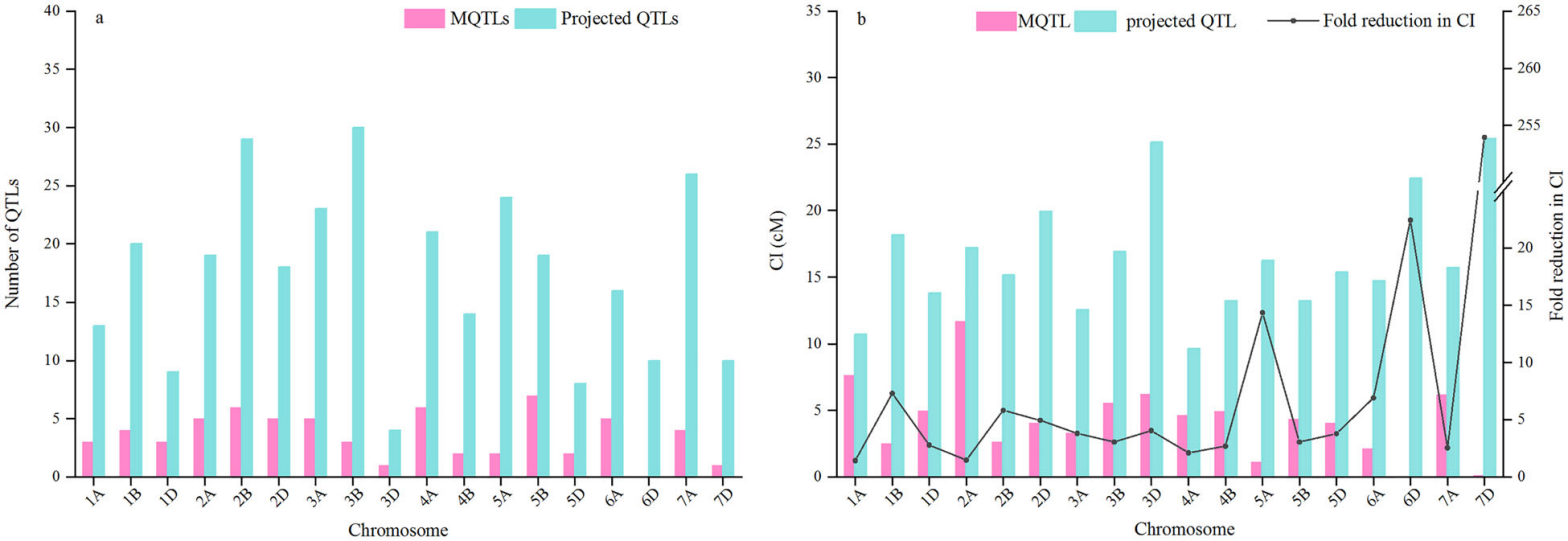
The information of QTL and MQTL used in this study. (a)The distribution of projected QTLs and MQTLs on chromosomes. (b) The reduction degree of QTL confidence interval (CI, 95%) after meta‐QTL analysis. The pink and blue bars represent the average CI length (cM) of MQTL and projected QTL on chromosome, respectively, and the broken line represents the reduction folds of the QTL CI length. MQTL, meta‐QTL; QTL, quantitative trait loci.

Reported QTLs for grain quality in wheat were from 41 previous studies published between 2003 and 2021 (Table S1). They were identified in 39 biparental mapping populations, including 32 RILs, seven DH, and two F_2_ populations. In total, we found 508 original QTLs for grain quality of wheat. Among these 508 QTLs, 46 QTLs were for PC, 241 QTLs were for GPC, 35 QTLs were for FPC, 70 QTLs were for WGC, and 116 QTLs were for TW. These initial 508 QTLs were distributed across all 21 chromosomes belonging to seven homeologous groups (1–7) and three sub‐genomes (A, B, and D), ranging from nine QTLs on 3D to 55 QTLs on 1B. The number of QTLs in the three sub‐genomes also varied, with 164 QTLs (32.28%) in sub‐genome A, 225 QTLs (44.29%) in sub‐genome B, and 119 QTLs (23.43%) in sub‐genome D. Furthermore, the initial CI of these QTLs ranged from 0.01 to 105.73 cM, with an average of 16.545 cM. Of the 508 QTL, 42.13% (214) had an initial CI of less than 10 cM, and 67.72% (344) had an initial CI of less than 20 cM. Accordingly, individual PVE ranged from 0.07% to 46.27%, with an average of 9.59%. Only 33.86% (172) of loci had PVE values greater than 10%, indicating that most loci were secondary QTL (Table S2). Of the 508 QTLs initially identified, only 313 (61.61%) loci were successfully mapped to the QTL consensus map (Figure S1). The remaining 195 QTLs were eliminated, due to the unavailability of common markers between the consensus and original linkage maps and the large CI of the original QTL. The 313 QTLs were distributed on all 21 chromosomes except 4D, 6B, and 7B, and the number of QTLs on each chromosome was also significantly different. More than 25 QTLs were found on chromosomes 2B (29), 3B (30), and 7A (26), whereas fewer than 10 QTLs were found on chromosomes 1D (9), 3D (4), and 5D (8). These results showed that QTLs related to grain quality traits in wheat were more distributed on A (142/313, 45.37%) and B (112/313, 35.78%) sub‐genomes, but less distributed on D (59/313, 18.85%) sub‐genome (Figure 1a). The initial CI of these QTLs ranged from 0.01 to 44.51 cM, with an average of 11.13 cM. Of the 313 QTLs, 54.63% (171) had an initial CI of less than 10 cM, and 82.11% (257) had an initial CI of less than 20 cM (Figure 1b). Accordingly, individual PVE ranged from 1.00% to 33%, with an average of 9.6%.

### 3.2 Identification of MQTL for grain quality traits

**FIGURE 2:**
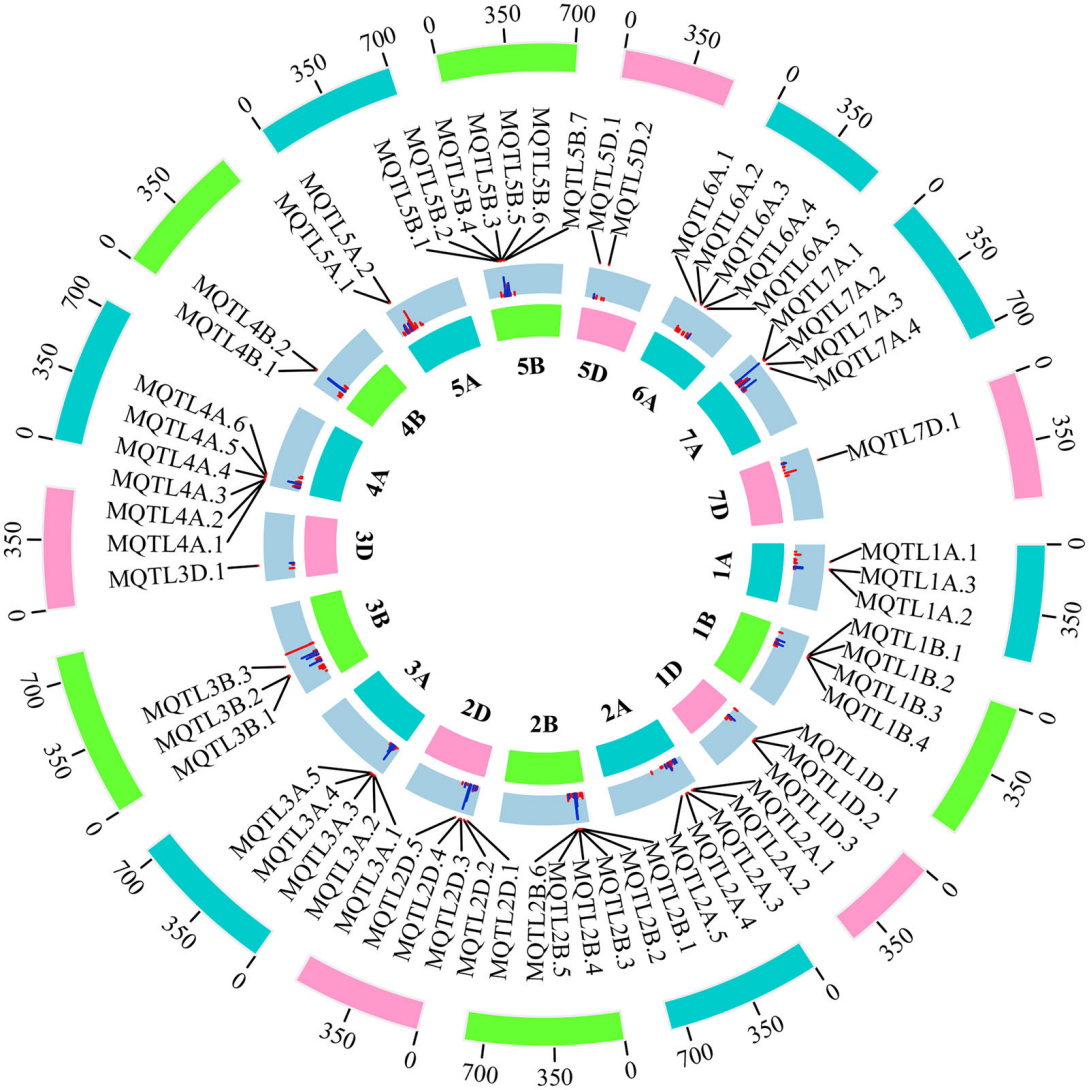
Chromosome distribution of 64 MQTLs for quality traits by MQTL analysis. The circles from outside to inside represent the chromosome length (Mb), the PVE (%) of the projected QTLs (red) and the number of included MQTLs (blue), the position of the MQTLs, and the genetic maps of the MQTLs. MQTL, meta‐QTL; PVE, phenotypic variance explained; QTL, quantitative trait loci.

Of the 313 projected QTLs, 64 MQTLs were finally identified based on the minimum model value and at least two overlapping original QTLs (Figure 2). Most MQTLs were associated with at least two different traits, reflecting the multi‐trait effects of grain quality formation. Among the 64 MQTLs, 10, 55, and 12 MQTLs contained QTLs for PC, GPC, and FPC, respectively; 22 and 24 MQTLs contained QTLs for WGC and TW, respectively; and 16 and nine MQTLs contained QTLs for GPC, WGC and GPC, FPC, respectively (Table 1). The 95% CI of the obtained MQTL ranged from 0.07 to 25.72 cM, with an average of 4.47 cM, which was 2.49 times less than that of the projected QTLs (11.13 cM) (Figure 1b), indicating that the mapping of these MQTLs was more accurate. The distribution of MQTLs on chromosomes was also different. For example, there were more than five MQTLs on chromosomes 2B, 4A, and 5B, whereas only one or two MQTLs were mapped on chromosomes 3D, 6D, and 7D (Figure 1a). The physical locations of these 64 MQTLs were determined by querying the multi‐omics database for wheat, and their physical intervals ranged from 0.45 to 239.01 Mb. Five core MQTLs, such as MQTL1A.1, MQTL1A.2, MQTL2A. 5, MQTL2D. 2, and MQTL5B.6 were positioned with a closer genetic distance of less than 1.0 cM, physical intervals of less than 20 Mb, and at least two overlapping QTL projections (Table 1). Thus, the selection criteria for further search for putative candidate genes were met.

**TABLE 1:** Summary of the 64 MQTLs detected in the present study

MQTL	Traits	No. of QTLs	Position (cM)	Confidence interval (cM)	Interval (cM)/(Mb)	Flanking marker	Physicial position (bp)
MQTL1A.1	GPC, WGC	2	150.52	150.45−150.59	0.14/3.77	wPt‐6709‐wsnp_Ex_c2868_5293485	3416736‐7184821
MQTL1A.2	GPC, FPC	4	219.04	218.615−219.465	0.85/3.88	Xcfd59‐BS00003758_51	159518846‐163393979
MQTL1A.3	GPC, FPC	4	228.46	217.47−239.45	21.98/52.79	IAAV2072‐Xbcd265	482278982‐535064611
MQTL1B.1	GPC, FPC	2	154.99	152.87−157.11	4.24/43.72	Xwpt‐4655‐Xwpt‐1363	193285394‐237002043
MQTL1B.2	GPC, TW	2	164.02	162.955−165.085	2.13/78.68	Xgwm18‐Tdurum_contig63991_280	222577490‐301257636
MQTL1B.3	GPC, TW	4	168.95	167.295−170.605	3.31/38.56	Xpsr949‐wPt‐2257	118128799‐156687025
MQTL1B.4	GPC, TW	2	179.62	179.495−179.745	0.25/1.96	Xwpt‐4532‐wPt‐4688	652297531‐654256761
MQTL1D.1	WGC, TW	4	97.86	97.08−98.64	1.56/7.89	BS00079095_51‐RAC875_c87321_108	408207062‐416101167
MQTL1D.2	WGC	2	115.27	112.36−118.17	5.81/10.83	wPt‐8854‐Xcdo312	383443597‐394278494
MQTL1D.3	GPC, TW	2	126.1	122.29−129.91	7.62/29.06	Kukri_c20062_389‐wmc36	379455295‐408511208
MQTL2A.1	GPC, PC	3	60.69	53.71−67.67	13.96/11.7	Xwms445‐IAAV1698	682622713‐694451138
MQTL2A.2	GPC, PC	3	84.38	71.52−97.24	25.72/22.01	wsnp_Ex_c7829_13320760‐RAC875_c13390_174	693293477‐715303241
MQTL2A.3	GPC, PC	2	114.48	113.185−115.775	2.59/17.18	wPt‐6245‐Xgwm359	11023629‐28201911
MQTL2A.4	GPC, TW	4	137.64	129.495−145.785	16.29/3.37	GENE‐1246_393‐GENE‐1075_265	68254790‐71623112
MQTL2A.5	GPC, WGC	3	173.82	173.775−173.865	0.09/3.82	wsnp_Ex_c2426_4542393‐Xgwm294a	712721102‐716545601
MQTL2B.1	GPC, WGC	2	77.29	74.41−80.17	5.76/8.17	Xpsr126‐Tdurum_contig23273_426	176413376‐184672580
MQTL2B.2	GPC, WGC, TW	5	87.35	85.93−88.77	2.84/7.26	wsnp_Ra_c2842_5399988‐BobWhite_c8113_532	202917363‐210259697
MQTL2B.3	GPC, WGC	4	93.93	93.335−94.525	1.19/6.05	Excalibur_c33221_681‐wsnp_Ex_c4024_7278036	736408809‐742525456
MQTL2B.4	GPC, FPC	8	96.92	95.125−98.715	3.59/23.68	Excalibur_c65341_303‐BS00063905_51	695581355‐719520773
MQTL2B.5	GPC, WGC, FPC	8	101.48	100.95−102.01	1.06/3.53	BS00063905_51‐RAC875_c2176_3046	728365134‐731891291
MQTL2B.6	GPC	3	107.77	107.17−108.37	1.2/2.69	wsnp_Ra_c54256_57628288‐Tdurum_contig41990_1324	706730243‐709425239
MQTL2D.1	FPC, TW	3	39.36	36.635−42.085	5.45/6.52	Excalibur_c14317_401‐Kukri_c74165_204	571075930‐577598871
MQTL2D.2	GPC	3	68.88	68.83−68.93	0.1/4.03	Xbcd1970‐Xwmc111	10331802‐14362953
MQTL2D.3	GPC, FPC	6	97.32	95.87−98.77	2.9/25.5	Xbcd111‐Xbarc11	441567951‐467066837
MQTL2D.4	GPC, FPC, PC	8	109.45	107.17−111.73	4.56/33.39	Kukri_c4949_261‐GENE‐0581_156	16493061‐49881785
MQTL2D.5	GPC, TW, PC	3	152.7	149.105−156.295	7.19/14.38	Kukri_c365_345‐Ex_c67599_547	635950371‐650326831
MQTL3A.1	GPC	2	51.91	49.885−53.935	4.05/10.25	Xwmc532‐Xcdo395	22351484‐32716614
MQTL3A.2	GPC	3	61.66	60.445−62.875	2.43/13.94	Tdurum_contig42496_1426‐Tdurum_contig55335_316	145259596‐159349782
MQTL3A.3	GPC	6	72.35	71.2−73.5	2.3/2.9	wsnp_Ex_rep_c69864_68824236‐Tdurum_contig98188_239	569423933‐572322783
MQTL3A.4	GPC, FPC	4	85.73	83.055−88.405	5.35/14	BS00022968_51‐wsnp_Ku_c5359_9530161	730279092‐744436234
MQTL3A.5	GPC, FPC	3	95.6	94.39−96.81	2.42/19.1	Xcdo113‐wsnp_Ex_c4923_8767234	611970623‐631281663
MQTL3B.1	GPC, WGC, PC	5	208.4	205.85−210.95	5.1/44.91	Xwmc527‐Tdurum_contig67693_344	540209527‐585606294
MQTL3B.2	GPC, EGC	3	214.62	213.695−215.545	1.85/29.57	wsnp_Ku_c29429_39332178‐XwPt‐6945	40245341‐69816299
MQTL3B.3	TW, PC	6	302.76	297.87−307.65	9.78/17.74	Xwpt‐5704‐IAAV4641	780812762‐798550682
MQTL3D.1	FPC, TW	3	146.17	143.05−149.29	6.24/23	Xgwm314‐Xgwm3b	556892544‐579890993
MQTL4A.1	GPC, TW	3	37.83	37.63−38.03	0.4/179.65	wPt‐7524‐Xwmc420	356604363‐538217416
MQTL4A.2	GPC, TW	2	39.05	38.41−39.69	1.28/23.38	Xbarc206‐TA004056‐0809	80095873‐103726346
MQTL4A.3	GPC	2	43.15	43.025−43.275	0.25/4.82	IAAV1476‐wsnp_BF474615A_Ta_1_1	577048567‐581869308
MQTL4A.4	GPC	2	63.02	61.405−64.635	3.23/10.52	Xcdo545‐wPt‐7168	705504155‐716137490
MQTL4A.5	GPC	5	76.88	75.885−77.875	1.99/10.6	wPt‐2983‐wPt‐2983	710051074‐720769068
MQTL4A.6	GPC, TW	2	100.13	94.58−105.68	11.1/0.45	wPt‐3506‐P40/M60‐221	720433311‐724879129
MQTL4B.1	GPC, WGC, TW	7	119.62	117.69−121.55	3.86/14.46	Xwms251‐wPt‐0872	568556138‐583020741
MQTL4B.2	GPC, TW	7	126.9	123.88−129.92	6.04/33.17	Xwpt‐1505‐Xbcd1265	434103909‐467271368
MQTL5A.1	GPC, TW	4	71.77	71.13−72.41	1.28/69.82	Xbarc141‐BS00096756_51	468935982‐538757186
MQTL5A.2	GPC, YW	2	81.13	80.64−81.62	0.98/36.3	Xbarc151‐wsnp_Ex_rep_c109532_92292121	558339994‐595037357
MQTL5B.1	GPC	2	151.9	147.31−156.49	9.18/44.81	XwPt‐1951‐Kukri_c98694_120	355236226‐400533268
MQTL5B.2	GPC, WGC, TW	3	162.31	161.51−163.11	1.6/13.11	Excalibur_c631_219‐Tdurum_contig31131_198	404187320‐417443595
MQTL5B.3	GPC, WGC, TW	6	169.27	167.635−170.905	3.27/8.93	Kukri_c57830_63‐BobWhite_rep_c57634_383	425671696‐434698859
MQTL5B.4	GPC, WGC, TW	7	169.27	166.125−172.415	6.29/35.63	Kukri_c57830_63‐BS00065895_51	425671696‐461298315
MQTL5B.5	GPC, WGC, TW	3	173.52	172.37−174.67	2.3/17.9	BS00065895_51‐RAC875_c24376_704	461298315‐479197716
MQTL5B.6	GPC, PC	4	193.95	193.895−194.005	0.11/19.59	RFL_Contig658_1166‐RAC875_c34910_173	479322964‐499128463
MQTL5B.7	GPC, WGC, PC	5	205.39	201.575−209.205	7.63/28.17	Xbcd1024‐BobWhite_c24484_240	674460491‐702941592
MQTL5D.1	GPC	3	87.67	84.35−90.99	6.64/55.9	Xcmwg770‐Xcfd8	339906761‐396417971
MQTL5D.2	GPC, WGC	2	164.1	163.35−164.84	1.49/47.62	Xcfd2‐Xgdm133	517967022‐625231068
MQTL6A.1	GPC	2	178.99	177.79−180.19	2.4/4.51	XwPt‐7572‐Tdurum_contig51566_427	595765058‐600275928
MQTL6A.2	GPC, WGC	2	236.4	235.1−237.7	2.6/27.5	Xwpt‐665782‐wPt‐1661	586039612‐613838719
MQTL6A.3	GPC	2	239.06	238.7−239.42	0.72/5.21	Xwpt‐9690‐XwPt‐0139	21948603‐27159391
MQTL6A.4	WGC	2	277.55	275.395−279.705	4.31/6.74	wsnp_JD_c5872_7032077‐BobWhite_c3506_1184	602708877‐609452933
MQTL6A.5	GPC, TW	3	303.95	303.635−304.265	0.63/1.13	Xwpt‐6696‐BS00046261_51	608156056‐609288327
MQTL7A.1	GPC	9	107.23	105.63−108.83	3.2/1.08	RAC875_rep_c114991_493‐BS00022404_51	19006634‐20097232
MQTL7A.2	GPC, PC	2	123.07	117.965−128.175	10.21/70.11	wsnp_Ex_c13248_20898211‐Ku_c32860_825	57873877‐128745629
MQTL7A.3	TW, PC	5	161.78	156.475−167.085	10.61/7.31	Excalibur_c780_694‐Excalibur_c22708_625	1408093‐8802762
MQTL7A.4	GPC	6	205.26	204.875−205.645	0.77/2.93	wsnp_CAP7_c1860_917952‐Ku_c19745_892	709130217‐712058609
MQTL7D.1	WGC, TW	2	130.95	130.9−131	0.1/239.01	Xbarc214‐Xwmc630	172661083‐414282693

Abbreviations: GPC, grain protein content; MQTL, meta‐QTL; PC, protein content; QTL, quantitative trait loci; TW, test weight; WGC, wet gluten content.

### 3.3 Verification of MQTL by previously published GWAS

**FIGURE 3:**
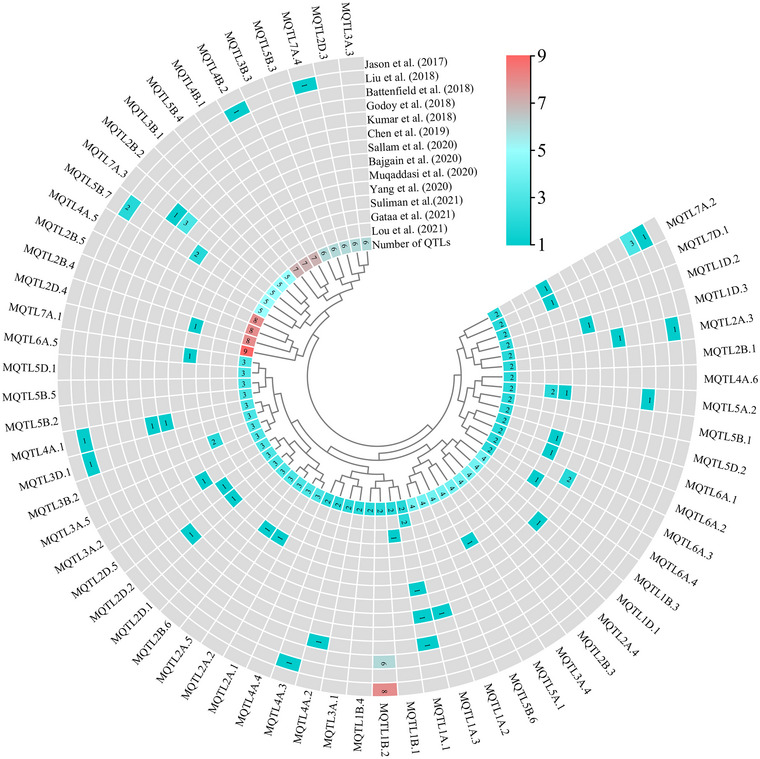
Validation of MQTL by MTAs for three quality traits in wheat from 13 different GWAS reports. The first circle of rectangles represents the initial QTL within MQTL, with the color from red to blue indicating the number of initial QTL from high to low. The remaining rectangles represent the colocation between MQTL and MTA, with the color from red to blue indicating the number of colocations from high to low. GWAS, genome‐wide association study; MTA, marker‐trait association; MQTL, meta‐QTL; QTL, quantitative trait loci.

The GWAS results on grain quality traits of wheat published in recent years were used to further verify the authenticity of the above MQTLs (Table S3). Considering the relatively long linkage disequilibrium decay distance of wheat (∼5 Mb), the MTAs obtained from GWAS near MQTLs in the 5 Mb physical region were associated with MQTLs. Therefore, by comparing the physical coordinates of MQTLs with the physical coordinates of MTAs for each trait, 31 of the 64 MQTLs (48.44%) were co‐located in at least one MTA (Figure 3). Some MQTLs were co‐located with multiple MTAs. Among them, 12 MQTLs that co‐localized with at least two MTAs, including MQTL1B.3 with 14 MTAs, followed by MQTL1A.1 and MQTL7A.2 with five MTAs. In addition, the physical coordinates of MQTL2D.4 (16.5–49.9 Mb) was also overlapped with the physical coordinates of MQTTL2D.3 (16.3–28.1Mb) identified in previous study (Saini et al., 2022).

### 3.4 Search for candidate genes within MQTL regions

Several strategies were used to identify candidate genes affecting wheat grain quality. First, the cloned genes controlling rice quality traits were searched in detail and 211 functional genes were identified. Then, their orthologous genes affecting quality traits in wheat were identified. Among them, there were 65 genes in 34 MQTL regions (Table S4), which were previously reported to regulate rice quality traits through multiple pathways, such as protein disulfide isomerase, starch synthase I, fragrance genes, seed disorder, amino acid transporter, histidine transporter, lysine‐histidine transporter and others. The orthologous wheat genes related to these rice genes also were identified by BLASTP. Of these, 79 candidate genes were found in 34 MQTL regions (with an average of two per MQTL). Several candidate genes in the MQTL regions had similar effects on quality‐related traits in wheat and rice. For instance, *TraesCS4A02G214200* (MQTL4A.1) and *PDIL1* affected PC, *TraesCS7A02G120300* (MQTL7A.2) and *OsSSI* affected starch content, and *TraesCS7D02G309400* (MQTL7D.1) and *OsHT* affected amino acid content. This result also indicated that the functions of the putative candidate genes are relatively conserved in rice and wheat. Moreover, these putative candidate genes have a high degree of confidence because their orthologous genes have been thoroughly investigated for their functions in rice quality traits. Second, 516 putative candidate genes were found among the five core MQTLs (MQTL1A.1, MQTL1A.2, MQTL2A. 5, MQTL2D. 2, and MQTL5B.6) (Table S5), and 625 candidate genes were identified by calculating the peak value of the remaining 25 MQTLs with longer CI (Table S6). A total of 1217 putative candidate genes (excluding duplicate genes) were identified in 64 MQTL regions, with an average of 19.02 candidate genes per region. Among these putative candidate genes, 66 were genes for proteins with F‐box family domains (such as *TraesCS3D02G465200, TraesCS3D02G464100*, and *TraesCS3D02G465400* on MQTL3D.1), 27 were genes for cytochrome P450 proteins (such as *TraesCS2D02G030700, TraesCS2D02G030000*, and *TraesCS2D02G023700* on MQTL2D.2), 14 were involved in NAD domain‐rich proteins (such as *TraesCS6A02G397200, TraesCS6A02G395600*, and *TraesCS6A02G397300* genes on chromosome 6A), four were involved in gamma‐gliadin formation (*TraesCS1A02G007200, TraesCS1A02G007700, TraesCS1A02G007400*, and *TraesCS1A02G007300*) and three genes were involved in the formation of the low molecular weight glutenin subunit (*TraesCS1A02G008000, TraesCS1A02G010900*, and *TraesCS1D02G317300*).

**FIGURE 4:**
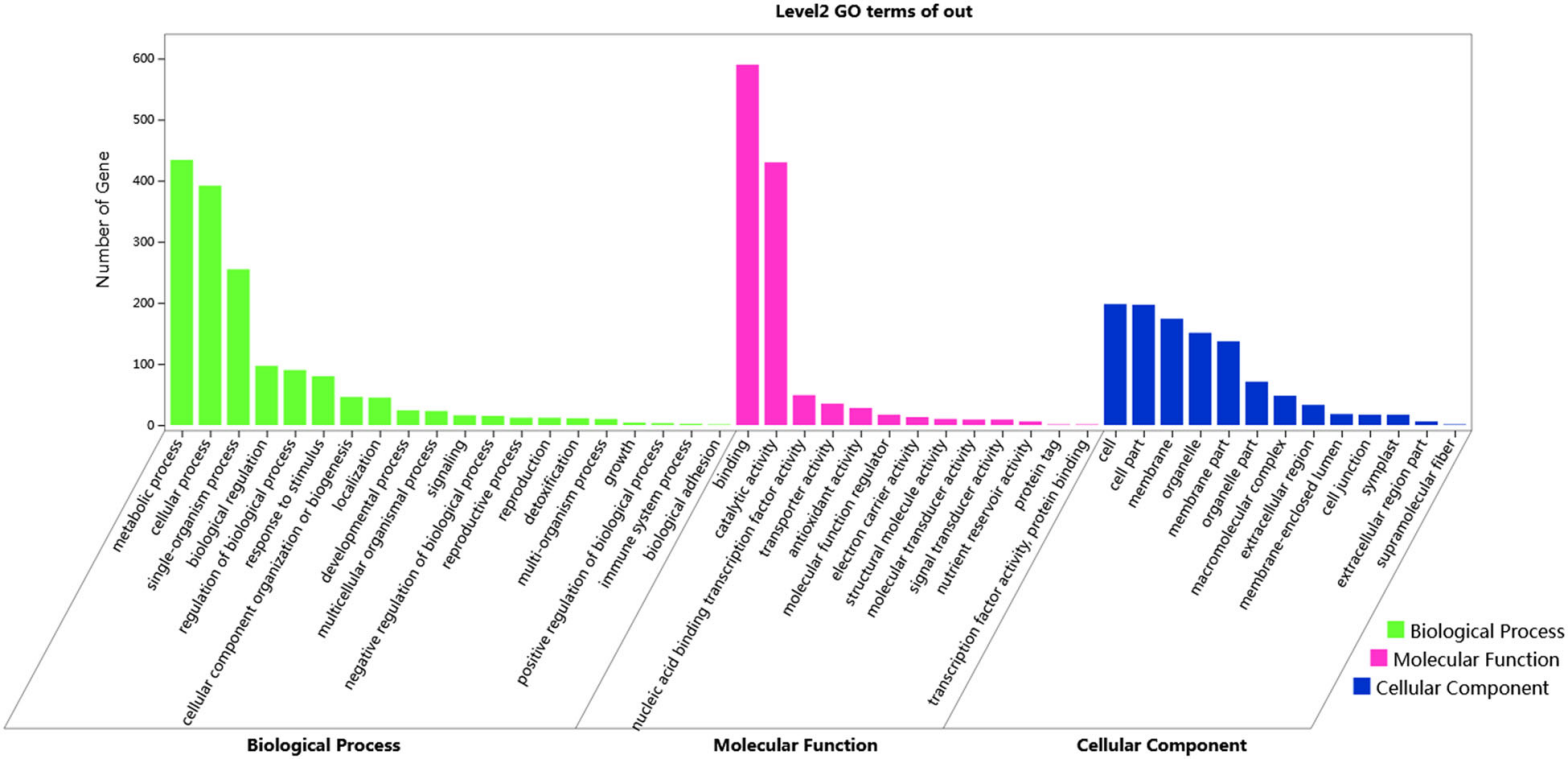
Level 2 gene ontology (GO) terms for all putative candidate genes from MQTL regions. MQTL, meta‐QTL.

**FIGURE 5:**
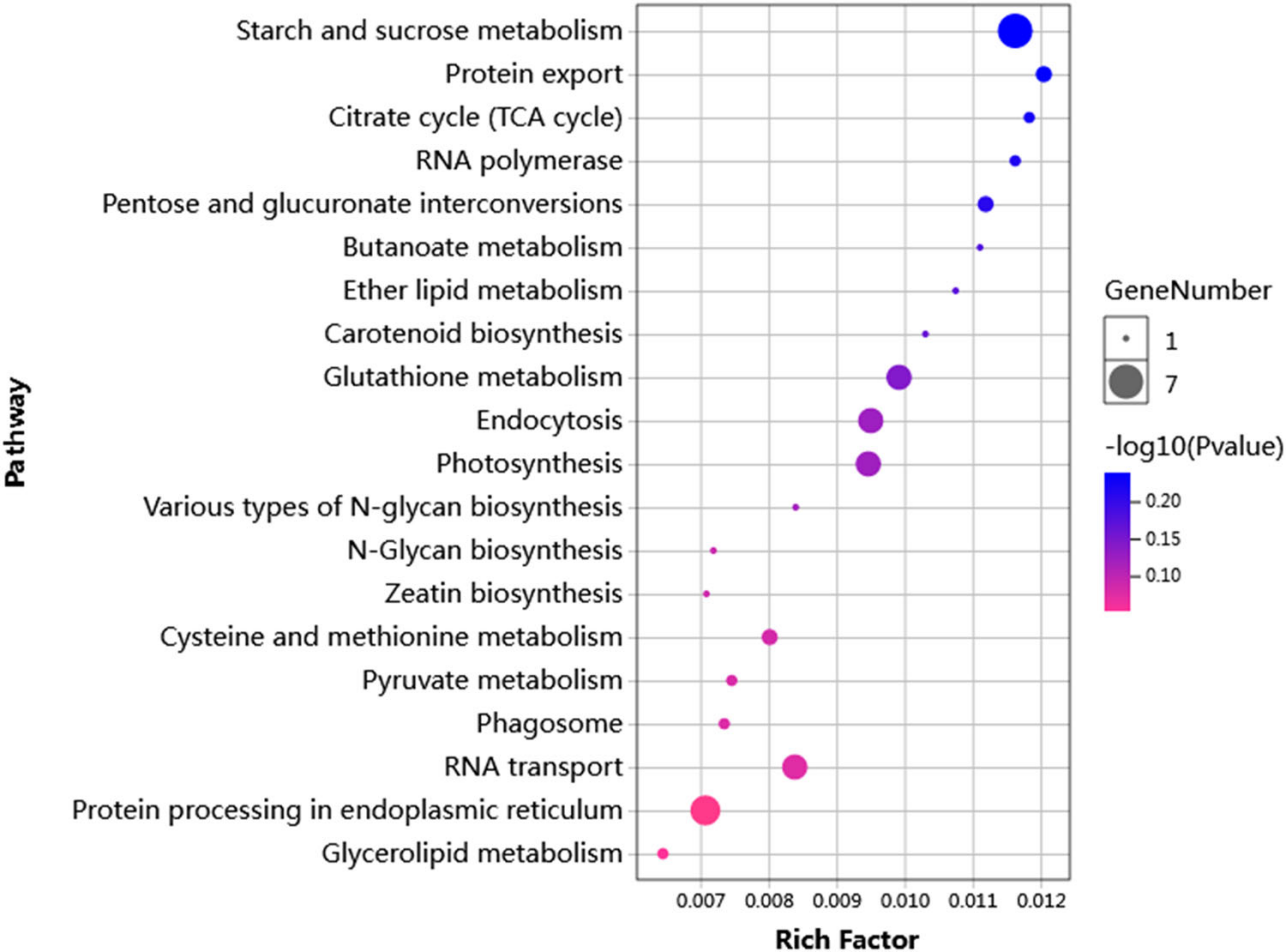
Twenty of all KEGG enrichment pathways for all genes from MQTL regions. Enrichment factors are shown on the horizontal axis and paths are shown on the vertical axis. Different colors represent different *p‐*value. From magenta to blue, the *p‐*value range from large to small, and the enrichment degree is more and more significant. The size of the circle represents the number of genes enriched in this pathway. KEGG, Kyoto Encyclopedia of Genes and Genomes; MQTL, meta‐QTL.

Lastly, GO and KEGG pathway enrichment analysis were used to obtain the functional classification of these genes, and they were mainly associated with biological processes (20 subfunctions), molecular functions (13 subfunctions), and cellular components (13 subfunctions) (Figure 4). The most enriched GO terms related to biological processes were metabolic processes (433/1217, 35.58%) and cellular processes (391/1217, 32.13%). The most enriched GO terms related to molecular function involved binding (588/1217, 48.32%) and catalytic activity (429/1217, 35.25%). As for the cellular component, the genes were mainly enriched in the cell (197/1217, 16.19%) and in the cell part (196/1217, 16.11%). In the KEGG metabolic pathways, some of these genes were strongly involved in quality‐related metabolic pathways, such as starch and sucrose metabolism, protein export, and citrate cycle (TCA cycle) (Figure 5).

### 3.5 In silico transcriptome assessments of candidate genes

**FIGURE 6:**
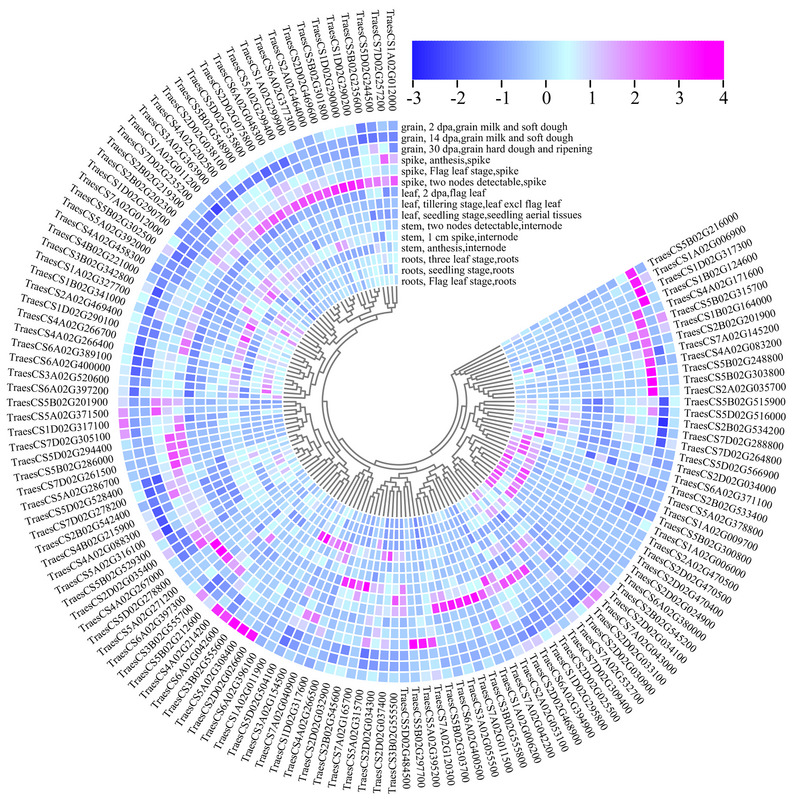
Expression characteristics of 135 candidate genes in five tissues. From blue to magenta, the expression of the gene in the relevant tissue ranges from low to high. dpa, day post anthesis.

In silico expression analysis showed that 135 putative candidate genes were highly and specifically expressed in grain, spike, leaf, and root (TPM > 2) (Figure 6, Table 2). The expression patterns of these putative candidate genes could be further divided into three classes. The putative candidate genes of class I were mainly expressed in the grain at the 14 dpa and 30 dpa stages. The putative candidate genes of class II were mainly expressed in the leaf at the 2 dpa stage and in the roots at the three leaf and flag leaf stages. The putative class III candidate genes were strongly expressed in the spike at the flag leaf stage and in two nodes detectable stages. Moreover, the expression of some genes such as *TraesCS5B02G216000, TraesCS7A02G145200*, and *TraesCS4A02G267000* varied in the same tissue at different growth stages.

**TABLE 2:** Summary of 135 putative candidate genes with significant expression (TPM > 2) within MQTLs

MQTL	Candidate gene ID	Gene function annotation	Orthologue in rice
MQTL1A.1	TraesCS1A02G006000	Leucine‐rich repeat receptor‐like protein kinase family protein, putative	Os11g0514700
	TraesCS1A02G006200	Receptor protein kinase, putative	Os01g0149700
	TraesCS1A02G006900	Receptor protein kinase, putative	NA
	TraesCS1A02G009700	Histone‐lysine N‐methyltransferase	NA
	TraesCS1A02G011200	Serine/threonine‐protein kinase 19	Os07g0458800
	TraesCS1A02G011900	Protein kinase	NA
	TraesCS1A02G012000	Protein kinase	NA
MQTL1A.3	TraesCS1A02G299900	L‐type lectin‐domain containing receptor kinase S.6	Os05g0550700
	TraesCS1A02G327700	Single‐stranded DNA‐binding protein, mitochondrial	Os05g0509700
MQTL1B.1	TraesCS1B02G164000	NA	Os10g0463800
	TraesCS1B02G341000	Single‐stranded DNA‐binding protein, mitochondrial	Os05g0509700
MQTL1B.2	TraesCS1B02G124600	Pyrophosphate‐energized vacuolar me‐mbrane proton pump 1	Os05g0156900
MQTL1D.1	TraesCS1D02G317100	Histone deacetylase	Os07g0602200
	TraesCS1D02G317300	High molecular weight glutenin subunit	Os05g0499100
	TraesCS1D02G317600	Protein kinase family protein	Os05g0498900
MQTL1D.2	TraesCS1D02G290000	Histone H4	Os05g0466600
	TraesCS1D02G290100	Histone H4	Os05g0462700
	TraesCS1D02G290200	Histone H4	Os05g0466600
	TraesCS1D02G290700	Calcium‐dependent protein kinase	Os05g0467000
MQTL1D.3	TraesCS1D02G295800	Receptor protein kinase, putative	Os05g0471000
MQTL2A.2	TraesCS2A02G464000	Remorin 4.1	Os04g0620200
MQTL2A.3	TraesCS2A02G035700	Arginase 1, mitochondrial	Os04g0106300
	TraesCS2A02G053100	G‐type lectin S‐receptor‐like serine/th‐reonine‐protein kinase LECRK2	Os04g0201900
MQTL2A.5	TraesCS2A02G469400	Histone H4	Os02g0684500
	TraesCS2A02G470500	Leucine‐rich repeat receptor‐like protein kinase family protein	NA
MQTL2B.1	TraesCS2B02G201900	Cytochrome P450 78A5	Os07g0603700
	TraesCS2B02G202300	Protein LONGIFOLIA 1	Os07g0603300
MQTL2B.2	TraesCS2B02G219300	Protein BZR1 homolog 1	Os07g0580500
MQTL2B.3	TraesCS2B02G542400	APETALA2‐like protein 5	Os04g0649100
	TraesCS2B02G545200	Tryptophan decarboxylase 1	Os08g0140300
	TraesCS2B02G545600	Protein TSS	Os04g0645100
MQTL2B.5	TraesCS2B02G533400	Serine/threonine‐protein kinase	Os04g0658700
	TraesCS2B02G534200	NADPH–cytochrome P450 reductase	Os04g0653400
MQTL2D.1	TraesCS2D02G468900	Starch synthase family protein	Os04g0624600
	TraesCS2D02G469600	Histone H4	Os10g0539500
	TraesCS2D02G470400	Leucine‐rich repeat receptor‐like protein kinase family protein	NA
	TraesCS2D02G470500	Leucine‐rich repeat receptor‐like protein kinase family protein	NA
MQTL2D.2	TraesCS2D02G024900	Receptor‐like protein kinase	NA
	TraesCS2D02G025500	Receptor‐like protein kinase	Os11g0225000
	TraesCS2D02G026600	Protein kinase family protein	NA
	TraesCS2D02G030900	Glyoxylate reductase/hydroxypyruvate r‐eductase	Os04g0107500
	TraesCS2D02G032900	NAD(P)‐binding Rossmann‐fold superfa‐mily protein	Os01g0367100
	TraesCS2D02G033100	Chitinase	Os05g0399300
	TraesCS2D02G034000	Glyoxylate reductase/hydroxypyruvate r‐eductase	Os04g0107500
	TraesCS2D02G034100	Glyoxylate reductase/hydroxypyruvate r‐eductase	Os04g0107300
MQTL	Candidate gene ID	Gene function annotation	Orthologue in rice
	TraesCS2D02G034300	Glyoxylate reductase/hydroxypyruvate r‐eductase	Os04g0107200
	TraesCS2D02G035400	3‐ketoacyl‐CoA thiolase	Os09g0252100
	TraesCS2D02G037400	Beta‐glucosidase	Os04g0513400
	TraesCS2D02G038100	Histone acetyltransferase	Os01g0246100
MQTL2D.4	TraesCS2D02G075800	NAD‐dependent protein deacetylase SRT1	Os04g0271000
MQTL3A.1	TraesCS3A02G055500	Sulfoquinovosyl transferase SQD2	Os01g0142300
MQTL3A.2	TraesCS3A02G154500	NA	Os01g0229300
MQTL3A.4	TraesCS3A02G520600	Bifunctional polymyxin resistance arnA protein	Os01g0969100
MQTL3A.5	TraesCS3A02G363900	BEL1‐like homeodomain protein 9	Os01g0848400
MQTL3B.1	TraesCS3B02G342800	Single‐stranded DNA‐binding protein, mitochondrial	Os05g0509700
MQTL3B.3	TraesCS3B02G555500	Protein kinase	Os01g0366300
	TraesCS3B02G555600	Protein kinase	Os01g0366300
	TraesCS3B02G555700	Protein kinase	Os01g0366300
	TraesCS3B02G555800	Protein kinase	Os01g0366300
MQTL4A.1	TraesCS4A02G171600	Probable glycerol‐3‐phosphate acyltran‐sferase 3	Os11g0679700
	TraesCS4A02G202500	Pentatricopeptide repeat‐containing protein At3g12770	Os11g0213500
	TraesCS4A02G214200	Protein disulfide‐isomerase	Os11g0199200
MQTL4A.2	TraesCS4A02G083200	Patatin‐like protein 6	Os03g0254400
	TraesCS4A02G088300	Vacuolar protein sorting‐associated pro‐ tein 9A	Os03g0262900
MQTL4A.3	TraesCS4A02G266400	NADH‐ubiquinone oxidoreductase subu‐ nit	Os03g0713400
	TraesCS4A02G266500	NADH‐ubiquinone oxidoreductase 75 K‐da subunit, mitochondrial	Os03g0713200
	TraesCS4A02G266700	Threonine dehydratase	Os03g0713000
	TraesCS4A02G267000	Phosphoglucosamine mutase	Os03g0712700
MQTL4A.6	TraesCS4A02G458300	protein kinase family protein	Os06g0714200
MQTL4B.2	TraesCS4B02G215900	Vacuolar protein sorting‐associated pro‐tein 9A	Os03g0262900
	TraesCS4B02G221000	Patatin‐like protein 7	Os03g0254400
MQTL5A.1	TraesCS5A02G271200	Tetraketide alpha‐pyrone reductase 1	Os09g0493500
	TraesCS5A02G286700	Squamosa promoter‐binding‐like pro‐tein 18	Os09g0507100
	TraesCS5A02G299400	Transcription factor RF2a	Os09g0516200
	TraesCS5A02G308400	Transcription factor RF2b	Os09g0520400
	TraesCS5A02G315700	Ras‐related protein RABA3	Os09g0527600
	TraesCS5A02G316100	Vacuolar protein sorting‐associated protein 22 homolog 1	Os09g0529700
MQTL5A.2	TraesCS5A02G371500	Protein YABBY 2	Os03g0650000
	TraesCS5A02G378800	Linoleate 9S‐lipoxygenase 2	Os03g0738600
	TraesCS5A02G392000	COBRA‐like protein 3	Os03g0754500
	TraesCS5A02G395200	Alpha‐1,4 glucan phosphorylase L isozy‐me, chloroplastic/amyloplastic	Os03g0758100
MQTL5B.1	TraesCS5B02G201900	Probable transcription factor RL9	Os09g0395300
	TraesCS5B02G212600	Auxin‐responsive protein SAUR76	Os08g0452500
MQTL5B.1	TraesCS5B02G216000	Probable indole‐3‐pyruvate monooxyg‐enase YUCCA11	Os01g0273800
MQTL5B.2	TraesCS5B02G235600	bZIP transcription factor TRAB1	Os09g0456200
MQTL5B.3	TraesCS5B02G248800	U‐box domain‐containing protein kinase family protein	NA
MQTL5B.5	TraesCS5B02G286000	Squamosa promoter‐binding‐like pro‐tein 18	Os09g0507100
MQTL5B.6	TraesCS5B02G297700	protein kinase family protein	Os09g0514200
	TraesCS5B02G300800	Cysteine synthase	Os04g0165700
	TraesCS5B02G301800	Histone H4	Os03g0119900
	TraesCS5B02G302500	GDP‐mannose‐3′,5′‐epimerase	Os11g0591100
MQTL	Candidate gene ID	Gene function annotation	Orthologue in rice
	TraesCS5B02G303700	NADP‐dependent alkenal double bond reductase	Os09g0459800
	TraesCS5B02G303800	NADP‐dependent alkenal double bond reductase	Os09g0459800
	TraesCS5B02G315700	UDP‐glucose 4‐epimerase	Os09g0526700
MQTL5B.7	TraesCS5B02G515900	Protein GLUTELIN PRECURSOR ACCUM‐ULATION 3	Os03g0835800
	TraesCS5B02G529300	Shaggy‐related protein kinase delta	Os03g0841800
	TraesCS5B02G548900	CTD small phosphatase‐like protein 2	Os03g0850100
MQTL5D.1	TraesCS5D02G244500	bZIP transcription factor TRAB1	Os09g0456200
	TraesCS5D02G278800	Tetraketide alpha‐pyrone reductase 1	Os09g0493500
	TraesCS5D02G294400	Squamosa promoter‐binding‐like protei‐n 18	Os09g0507100
MQTL5D.2	TraesCS5D02G484500	Glucose‐1‐phosphate adenylyltransfera‐se large subunit 1, chloroplastic/amylo‐plastic	Os03g0735000
	TraesCS5D02G504100	Nonspecific phospholipase C1	Os03g0826600
	TraesCS5D02G516000	Protein GLUTELIN PRECURSOR ACCUM‐ULATION 3	Os03g0835800
	TraesCS5D02G528400	Shaggy‐related protein kinase delta	Os03g0841800
	TraesCS5D02G535800	CTD small phosphatase‐like protein 2	Os03g0850100
	TraesCS5D02G566900	Protein DETOXIFICATION 56	Os03g0858800
MQTL6A.2	TraesCS6A02G371100	Betaine aldehyde dehydrogenase 2	Os08g0424500
	TraesCS6A02G377300	Protein G1‐like6	Os02g0811000
	TraesCS6A02G380000	Phosphate transporter PHO1‐2	Os02g0809800
	TraesCS6A02G389100	NADH dehydrogenase	Os02g0816800
MQTL6A.3	TraesCS6A02G042600	Luminal‐binding protein 4	Os02g0115900
	TraesCS6A02G048300	Putative pentatricopeptide repeat‐conta‐ining protein At1g53330	Os05g0207200
MQTL6A.5	TraesCS6A02G394900	Protein kinase	Os02g0819600
	TraesCS6A02G396100	Serine/threonine‐protein kinase	Os02g0777400
	TraesCS6A02G397200	NADH‐ubiquinone oxidoreductase chain 6	NA
	TraesCS6A02G397300	NADH‐ubiquinone oxidoreductase chain 1	BAC19852
	TraesCS6A02G400000	Aconitate hydratase	Os08g0191100
	TraesCS6A02G400500	Leucine‐rich repeat receptor‐like protein kinase family protein	NA
MQTL7A.1	TraesCS7A02G040900	Sucrose synthase	Os03g0401300
	TraesCS7A02G042200	Receptor protein kinase, putative	Os02g0553000
	TraesCS7A02G043000	Shikimate kinase	Os06g0114650
MQTL7A.2	TraesCS7A02G120300	Starch synthase 1, chloroplastic/amylop‐lastic	Os06g0160700
	TraesCS7A02G145200	NA	Os06g0181300
	TraesCS7A02G165700	Anthocyanin regulatory C1 protein	Os06g0221000
MQTL7A.3	TraesCS7A02G011500	Leucine‐rich receptor‐like protein kinase family protein	NA
	TraesCS7A02G012000	6‐phosphogluconate dehydrogenase, d‐ecarboxylating	Os06g0111500
MQTL7A.4	TraesCS7A02G532700	Protein kinase	NA
MQTL7D.1	TraesCS7D02G235200	Alpha‐glucan water dikinase, chloropla‐stic	Os06g0474866
	TraesCS7D02G257200	Pentatricopeptide repeat‐containing pr‐otein At5g18390, mitochondrial	Os08g0525500
	TraesCS7D02G261500	Squamosa promoter‐binding‐like protei‐n 16	Os08g0531600
	TraesCS7D02G264800	OVARIAN TUMOR DOMAIN‐containing deubiquitinating enzyme 1	Os08g0537800
	TraesCS7D02G278200	Protein‐L‐isoaspartate O‐methyltransfe‐rase	Os08g0557000
	TraesCS7D02G288800	Glycoprotein 3‐alpha‐L‐fucosyltransfer‐ase A	Os08g0472600
	TraesCS7D02G305100	Polycomb group protein FIE1	Os08g0137100
	TraesCS7D02G309400	Lysine histidine transporter 1	Os08g0127100

Abbreviations: MQTL, meta‐QTL; NA, not available; TPM, transcripts per million.

## 4 DISCUSSION

The discovery of molecular markers and advances in QTL mapping strategies have facilitated the identification of many QTLs controlling quality traits in wheat. However, many of these QTLs have low PVE values and large CI, so they were not suitable for marker‐assisted plant breeding (Saini et al., 2022). In the present study, we performed a meta‐analysis of 508 initial QTLs associated with three quality traits from 41 independent studies and found that these 508 initial QTLs were unevenly distributed across three genomes, with most QTLs on genomes A and B and the fewest on the D genome. The 95% CI of 64 MQTLs obtained in this study was 3.70 times lower than that of the original QTL studies. In a similar study, 141 MQTLs were detected with an average CI of 1.4 Mb, which was 8.8 times less than the CI (>12.1cM) of the original QTLs (Saini et al., 2022). Based on the released reference sequence of the Chinese Spring wheat genome, the 64 MQTLs detected in this study have unique physical positions, with physical intervals ranging from 0.45 to 239.01 Mb. Moreover, the distribution of MQTL was unequal across the different chromosomes, with the A and B genome carrying significantly more MQTLs than the D genome. The results were consistent with previous MQTL analyses of grain quality, yield, and yield‐related traits (Saini et al., 2022; Wang et al., 2015; Yang et al., 2021). It is possible that there are fewer QTLs for our target traits on the D sub‐genome. However, it is also possible that QTL detection is affected by the lower level of DNA polymorphisms present on the D sub‐genome. Because the D sub‐genome is a recent evolutionary addition to the hexaploid wheat genome, gene flow from *Aegilops tauschii* to cultivated wheat is limited, resulting in relatively low genetic variation (Kumar et al., 2012).

To validate the MQTL results based on previously GWAS reports, we found that 48.44% (31/64) of the 64 MQTLs identified in this study were co‐localized with MTAs identified by GWAS, which is consistent with previous studies (Aduragbemi et al., 2022; Yang et al., 2021) comparing MQTLs with MTAs. When MQTL were verified by more than one GWAS‐MTAs study and included many original QTLs from independent experiments, the MQTL examined in our study may be more stable and accurate. In this case, nine MQTLs were detected at least twice in 13 GWAS reports, of which MQTL1A.1, MQTL4A.1, MQTL5A.3, and MQTL7A.2 were detected three times. This further confirmed the reliability of the present MQTLs. The identification of these MQTLs provided a basis for the accurate search of quality‐affecting candidate genes that can facilitate molecular cloning and functional identification of genes related to grain quality in wheat.

Among the 64 MQTLs found in this study, five MQTLs were designated as core MQTLs. The interval of these core MQTLs had a relatively small genetic distance (*n* < 1.0cM), a smaller physical distance (*n* < 20Mb), and contained more original QTLs (*n* > 2) (Venske et al., 2019). These five core MQTL had a smaller CI (0.26cM), which was 17.19‐fold smaller than all MQTL (4.47cM) in this study. Moreover, four of the five core MQTLs were controlling two traits, for example, MQTL1A.1 (GPC, WGC), MQTL1A.2 (GPC, FPC), MQTL2A.5 (GPC, WGC), and MQTL5B.6 (PC, GPC). In addition, studies show that GPC and WGC, and GPC and FPC are highly positively correlated (*p* < 0.01) (Huang et al., 2006; Liu et al., 2017; Raman et al., 2009) with correlation coefficients of 0.74 and 0.92, respectively. Three of the five core MQTLs contained initial QTLs detected in quite diverse and varied segregating populations, such as MQTL5B.6, which consisted of four initial QTLs from three different populations, with an average PVE of 20.02% (Gonzalez‐Hernandez et al., 2004; Krishnappa et al., 2021; Yang et al., 2013). MQTL2A.5 was composed of three original QTLs from two different populations, with an average PVE of 7.84% (Cui et al., 2016; Peleg et al., 2009). MQTL1A.2 was composed of two initial QTLs from two different populations, with an average PVE of 6.58% (Raman et al., 2009; Sun et al., 2016). Interestingly, all five core MQTLs controlled for the same trait (GPC). The physical positions of the genes identified by previous GWAS, and meta‐analyses overlapped with the MQTL positions in the current study (Gudi et al., 2022; Yang et al., 2021). This also proves the reliability of the currently determined MQTLs to be used for map‐based cloning of quality traits in further studies.

Previous studies have shown that polyphenol oxidase (PPO) is the major cause of time‐dependent discoloration in raw wheat dough (Beecher et al., 2011). Its homologs *PPO‐A1, PP0‐A2, PPO‐D1*, and *PPO‐D2* were strongly expressed in grains and their physical location was found within the regions of MQTL2A.2 and MQTL2D.1 in the current study. The two genes *Traescs2D02G033900* and *Traescs2D02G468600* encoding PPO, were also identified in the interval of our MQTL2D.2 and MQTL2D.1. In addition, the early study showed that OsmtSSB1‐mediated mitochondrial function plays a critical role in determining subaleurone cell fate in rice (Li et al., 2021), and its related gene *TraesCS1A02G327700* was found in the region of MQTL5B.2 in this study.

As shown in the previous studies, MQTL regions correlate strongly with gene density in the genome (Kumar et al., 2020; Li et al., 2013; Maccaferri et al., 2015; Saini et al., 2022). For instance, they identified 15,772 gene models for yield, baking quality, and GPC (Quraishi et al., 2017) and 2533 gene models for rheological dough characteristics, nutritional properties, and processing quality (Gudi et al., 2022). In our study, 1217 genes were obtained in the interval of 64 MQTLs and selected for further analysis. Some of our putative candidate genes were strongly implicated in the pathways of starch and sucrose metabolism, protein export, and the TCA cycle (Figure 5). Starch and sucrose metabolisms play an important role in carbohydrate partitioning in the plants and have a major impact on wheat quality. For example, granule‐bound starch synthase I, which controls amylose content (Martin et al., 2004), and key enzymes that control amylopectin content, namely soluble starch synthase (Li et al., 2003), de‐branched enzyme, and branched enzyme (Crofts et al., 2017), have an impact on final quality by affecting processing quality.

In our study, the expVIP platform was used to identify 1217 putative candidate genes in the MQTL region. Only the putative candidate genes exhibiting more than two TPM were considered (Venske et al., 2019; Wagner et al., 2013), combined with some genes previously reported to affect grain quality traits in wheat. For instance, the *GPC, Glu‐1, Glu‐3*, and *Gli* genes, which encode NAD transcription factor, high and low molecular weight glutenin subunit (HMW‐GS/LMW‐GS), and gliadin, respectively, and are directly involved in grain protein formation (Krystkowiak et al., 2017; Plessis et al., 2013; Uauy et al., 2006). Grain protein is one of the three nutrients in food, which is of great significance to the color, aroma, and taste of food. We found a total of 135 putative genes with high and/or specific expression patterns in grains, ears, or leaves (TPM > 2) (Figure 6). These putative candidate genes showed tissue and development‐dependent expression patterns that can strongly influence grain quality traits. For example, *TraesCS1D02G317300*, encoding the high molecular weight glutenin subunit, was specifically expressed in grain 14 dpa. The content of HMW‐GS in wheat grains is low, accounting for only about 10% of the traditional storage protein but may explain about 60% of the variation in bread baking quality of wheat (He et al., 2005; Singh et al., 2016). *TraesCS2A02G035700* and *TraesCS2D02G034900*, encoding the arginase family, were specifically expressed in cereals. In rice, the homologous gene *OsARG* is the key enzyme of arginine metabolism. Loss of arginase activity in the *nglf‐1* mutant resulted in accumulation of arginine in panicles and increased seed formation rate, grain yield, and number of full grains (Ma et al., 2013). Studies have demonstrated that histone deacetylases (HDACs) play a key role in plant growth and development, including flower development (Li et al., 2011), seed development (Wu et al., 2000), root development (Xu et al., 2005), and cell proliferation (Nelissen et al., 2005) and cell death (Bourque et al., 2011; Huang et al., 2007) during organ growth (Dong et al., 2016). *TraesCS2D02G075800* encodes NAD‐dependent protein deacetylase and conserved HDACs family (Imai et al., 2000). These genes could be further investigated to reveal their possible role in wheat quality and their application in breeding programs. Although many genes related to wheat quality are rarely studied, their homologous genes in rice and *Arabidopsis* have been reported to potentially play key roles in plant growth and development. For example, some homologous genes in rice have been implicated in the regulation of rice quality traits, such as *OsmtSSB1, WTG1*, and *OspPLAIIIα* (Huang et al., 2017; Li et al., 2021; Liu et al., 2015). Other identified genes related to protein and sugar transport were also strongly expressed in the corresponding tissues, suggesting that these 135 putative candidate genes are most likely involved in the regulation of grain quality traits in wheat.

## 5 CONCLUSION

This study confirms the validity and reliability of the meta‐analysis method for understanding the genetic mechanisms underlying grain quality traits in wheat. The candidate genes we identified all have a direct or indirect effect on grain development and ultimately grain quality. The critical genes were involved in three metabolic pathways involving starch and sucrose metabolism, protein export, and the TCA cycle. Some of the genes had functions as previously reported for grain development and quality formation. The discovered MQTL regions and candidate genes can be used in marker‐assisted selection to improve grain quality in future breeding research programs.

## AUTHOR CONTRIBUTIONS

**Na Li**: Conceptualization; data curation; formal analysis; methodology; resources; software; validation; writing–original draft. **Yongping Miao**: Conceptualization; data curation; software. **Jingfu Ma**: Conceptualization. **Peipei Zhang**: Formal analysis; validation. **Tao Chan**: Formal analysis; validation. **Yuan Liu**: Formal analysis. **Zhuo Che**: Data curation; resources. **Fahimeh Shahinnia**: Writing–review and editing. **Delong Yang**: Methodology; supervision; writing–review and editing.

## CONFLICT OF INTEREST STATEMENT

The authors declare no conflicts of interest.

## Supporting information

Supplementary material

## Data Availability

The datasets used and/or analyzed during the current study are available from the corresponding author on reasonable request. Some of the data are shown in Supporting Information.
